# Prevalence of respiratory virus in symptomatic children in private physician office settings in five communities of the state of Veracruz, Mexico

**DOI:** 10.1186/s13104-015-1239-0

**Published:** 2015-06-25

**Authors:** Rosa M Wong-Chew, Marco A Espinoza, Blanca Taboada, Fernando E Aponte, María A Arias-Ortiz, Jesús Monge-Martínez, Rubén Rodríguez-Vázquez, Fidel Díaz-Hernández, Fernando Zárate-Vidal, José I Santos-Preciado, Susana López, Carlos F Arias

**Affiliations:** Unidad de Investigación en Medicina Experimental, Facultad de Medicina, Universidad Nacional Autónoma de México, Dr. Balmis 148, Colonia Doctores, CP 06726 Mexico, D.F. Mexico; Instituto de Biotecnología, Universidad Nacional Autónoma de México, Av. Universidad 2001, Colonia Chamilpa, 62210 Cuernavaca, Morelos Mexico; Colegio de Pediatría del Estado de Veracruz, Veracruz, Veracruz Mexico

**Keywords:** Respiratory virus infections, Community setting, Children

## Abstract

**Background:**

Acute respiratory tract infections are the leading cause of morbidity and mortality in children worldwide. Many studies have described the frequency of viruses in hospitalized patients, but studies describing the prevalence of viruses in the community setting are limited, particularly in developing countries, where most of the deaths from serious respiratory diseases occur. The aim of this study was to evaluate the diversity of respiratory viruses in the community setting using molecular diagnostic tools, as well as the clinical characteristics of respiratory viral infections in the general pediatric practice in Mexico.

**Methods:**

Children with respiratory tract infections attending private pediatric practices during a 10-month period in five cities of the state of Veracruz were included. Nasal swabs were taken and processed by a multiplex detection kit for 15 respiratory viruses.

**Results:**

525 children were included from July 2011 to May 2012; 44% were female, mean age was 45 months. The 3 most frequent clinical diagnosis were: rhinopharyngitis 68%, pharyngitis 18%, and 3.3% influenza-like illness. 71.5% of the samples were positive for virus. The five most frequent pathogens were respiratory syncycitial virus in 18.3% of the children, rhinovirus in 17.5%, influenza A 9.1%, adenovirus 7.2%, and enterovirus 3.4%, although all 15 viruses were detected; there were viral coinfections in 14.1%, and 28.5% of the samples were negative.

**Conclusions:**

A large proportion of respiratory infections in the community setting in Mexico was associated to viruses. Although testing for common respiratory pathogens in children with acute respiratory tract infections may lead to a better understanding of the role of viral pathogens in, and eventually to improvement in the management of, individual patients, additional prospective studies are required to study the need of routinely using such tests in general pediatric practices in resource-limited countries.

## Background

Respiratory tract infections continue to be the most frequent reason for health care visits and hospitalizations and are a leading cause of morbidity and mortality, particularly in young children. According to a report of the World Health Organization, there were 6.6 million deaths in 2012 in children under 5 years of age worldwide [1]. The highest mortality occurred in developing countries, where 15% of the deaths were caused by acute respiratory tract infections. Mexico reported more than 26 million acute respiratory tract infections in 2013 [2]. The Ministry of Health reports the annual incidence and prevalence, but the etiology of these pathologies is unknown.

Many studies have described the frequency of viruses in hospitalized patients, but studies describing the prevalence of viruses in the community setting are limited, particularly in developing countries. Previous reports in Mexico have evaluated the presence of at most seven viruses in a single study using different methods, such as viral culture, immunofluorescence, or single PCR. The majority of these studies have been carried out in hospital settings, which limits the available information about the pathogens causing respiratory infections in the community.

Newly developed commercial diagnostic assays based on real-time nucleic acid amplification, such as multiplex polymerase chain reaction (PCR) tests allow sensitive and specific detection of a broad panel of conventional and emerging viruses in respiratory tract specimens. The multiplex PCR assays are more sensitive than any other diagnostic method, including virus isolation in cell culture and antigen detection, and are being used around the world for the past few years [3]. These technological advances have changed the landscape of virus detection and provide the opportunity to better understand the epidemiology of respiratory viruses.

The aim of our study was to evaluate the diversity of respiratory viruses using new diagnostic tools in children with symptomatic respiratory tract infections in private physician office settings in five communities of the state of Veracruz, Mexico. This information contributes to the knowledge about the etiology of one of the most frequent diseases in our country, and one of the first cause for consult in the pediatric offices.

## Results

### Demographic characteristics and risk factors

525 children from private pediatric practices in 5 different municipalities of the state of Veracruz, Mexico, were included; 56% were male and the mean ± SE age was 45 ± 2 months, mean ± SE weight was 17 ± 0.7 kg, mean ± SE height was 93 ± 1 cm. There were statistically significant differences among the municipalities (Table 1): the oldest children were from Poza Rica and Minatitlán. The parental education was also different among the cities: the parents with the highest education were in Córdoba, Minatitlán, and Veracruz, although Córdoba had some parents with no formal education (3%). The socioeconomic level of the majority of the families was in the middle-income category, except for those attending the pediatric practice in the city of Veracruz where 36% reported high income. Some risk factors for respiratory tract infection were documented: asthma was present in 22%, allergic rhinitis in 25%, and incomplete vaccination scheme in 29% of the population (Table 1). Asthma was diagnosed by the attending physician according to Global Initiative for Asthma (GINA) in its report Global Strategy for Asthma Management and Prevention in children 5 years and younger [4]. Children under 3 years of age were not considered asthmatic.

**Table 1:** Demographic characteristics and risk factors of children 1–15 years old with respiratory tract infections in the community setting in the state of Veracruz, Mexico

	Córdoba	Tierra Blanca	Veracruz	Poza Rica	Minatitlán	Total	p
Demographic characteristics	n = 60	n = 74	n = 66	n = 208	n = 111	n = 519	
Male, n (%)	37 (60)	36 (48)	44 (67)	108 (52)	66 (59)	291 (56)	
Female, n (%)	23 (40)	38 (52)	22 (33)	100 (48)	45 (41)	228 (44)	0.1
Age in months (mean ± SE)	30 ± 4	32 ± 4	32 ± 5	57 ± 3	44 ± 4	45 ± 2	<0.005
Age in months (median, range)	16 (1–191)	16 (1–144)	16 (1–180)	43 (3–290)	24 (0–192)	26 (0–290)	<0.005
Weight (kg ± SE)	14.6 ± 2	13.7 ± 1	12 ± 1	21 ± 1	18 ± 1	17 ± 0.7	<0.005
Height (cm ± SE)	87 ± 3	87 ± 3	52 ± 1	102 ± 2	96 ± 3	93 ± 1	<0.005
Parental education	n = 60	n = 71	n = 18	n = 211	n = 111	n = 461	
None, n (%)	2 (3)	0	0	0	0	2 (0.4)	
Primary, n (%)	3 (5)	8 (11)	0	8 (4)	5 (4.5)	24 (5)	
Middle school, n (%)	3 (5)	20 (28)	2 (17)	36 (17)	14	75 (16)	0.001
High school, n (%)	13 (21)	20 (28)	4 (33)	76 (36)	32 (13)	142 (31)	
Bachelor, n (%)	39 (65)	22 (31)	6 (50)	90 (43)	57 (51)	213 (46)	
Postgraduate, n (%)	0	1 (2)	0	1 (0.5)	3 (3)	5 (1)	
Socioeconomic level	n = 60	n = 73	n = 64	n = 198	n = 110	n = 505	
Low income, n (%)	4 (6)	42 (57)	1 (1)	54 (37)	16 (15)	117 (23)	0.000
Middle income, n (%)	56 (93)	31 (42)	40 (62)	144 (73)	94 (85)	365 (73)	
High income, n (%)	0	0	23 (36)	0	0	23 (4)	
Risk factors	n = 57	n = 71	n = 66	n = 208	n = 110	n = 514	
Asthma, n (%)	3 (5)	34 (48)	3 (5)	27 (13)	28 (25)	97 (22)	
Allergic rhinitis, n (%)	4 (7)	13 (18)	17 (27)	35 (20)	58 (53)	127 (25)	0.000
Complete vaccination scheme, n (%)	8 (14)	61 (87)	33 (100)	204 (92)	44 (42)	333 (71)	

### Detection of viral pathogens and coinfections

**Figure 1:**
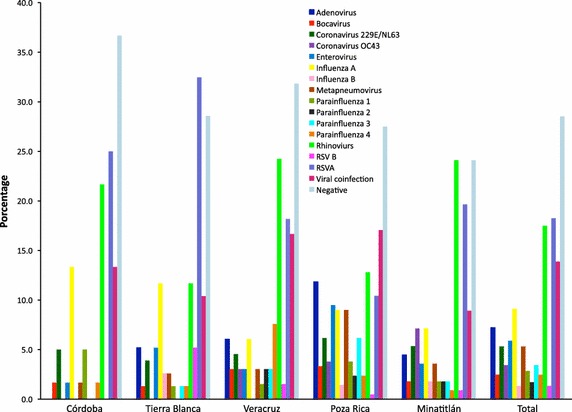
Frequencies of viral pathogens in five different municipalities of Veracruz. The percentage of each virus, considering their presence in both single and multiple infections, is shown for the five cities included in the study. The percentage of viruses in the total number of samples analyzed is also shown. Viral coinfection stands for the percentage of samples in which more than virus was found.

From the 525 nasal swabs tested by PCR, 71.5% were positive for the presence of viral nucleic acids of one or more respiratory virus, while 28.5% of the samples were negative. Among the viruses detected, considering both single and multiple infections, RSV-A and RV showed the highest frequency, being present in 18.3 and 17.5% of the samples analyzed, respectively, followed by FluA (9.1%), HuCoV OC43 + 229E/NL63 (8.7%), AdV (7.2%), EV (5.9%), HMPV (5.3%), PIV-3 (3.4%), PIV-1 (2.9%), PIV-4 (2.5%), HuBoV (2.5%), PIV-2 (1.7%), RSV-B (1.3%), and FluB (1.3%) (Figure 1).

In 73 of the 525 samples (13.9%) a viral coinfection was found. Sixty-three of these samples had a dual infection, with the combination of AdV/EV and RV/HuCoV 229/N63 being the more frequent. Eight children had triple virus infections, and two were infected simultaneously with four viruses (Table 2).

**Table 2:** Virus coinfections

	AdV	HuBoV	HuCoV^c^	HuCoV^b^	EV	Flu A	HuMPV	PIV-1	PIV-3	PIV-4	RV
Positive for two virus											
CoV OC43	2	1									
CoV 229E/NL63			1								
Enterovirus	4	1		1							
Influenza A	3	1									
Metapneumovirus			1	3		1					
PIV-1							1				
PIV-2	1		1		1	1					
PIV-3			1		2	1		1			
PIV-4		1				1	1				
Rhinoviurs	3			4	1	2		3	1	3	
RSV A	2	2		3				1	1	1	3
RSV B						1					
Positive for three viruses											
Adenovirus	RV	HuMPV	2								
CoV OC43	EV	HuCoV^b^	1								
Metapneumovirus	EV	HuBoV	1								
PIV-1	AdV	RV	1								
PIV-3	HuCoV^c^	RV	1								
Rhinoviurs	PIV-3	PIV-2	1								
Rhinoviurs	RSV A	EV	1								
Positive for four viruses											
Adenovirus	RV	RSV B	PIV-4	1^a^							
Adenovirus	RV	RSV A	EV	1							

^a^Numbers indicate the number of samples with the indicated combination of viruses.

^b^HuCoV 229E/NL63.

^c^CoV OC43.

### Seasonality

**Figure 2:**
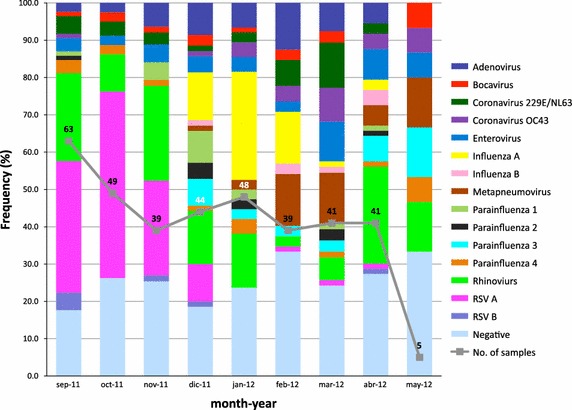
Seasonal prevalence of respiratory tract infections in the state of Veracruz. The frequency of the different viruses in each month of the study is shown. The data represents the percentage of each virus detected in both single and mixed infections in a given month, divided by the total number of viruses found in that month plus the number of negative samples of the month. Since viruses from both single and mixed infections were considered, the calculations mentioned above were done to adjust each month to 100% frequency. The *continuous line* indicates the number of samples analyzed each month.

Seasonality is well described for several viral respiratory pathogens [5, 6]. In our study there was a seasonal predominance for RSV-A from September to December, FluA was more frequent from December to February, FluB from December to April, and HMPV predominated from February to May. In contrast, RV, EV, AdV, and PIV-3 were present during all the study period (Figure 2).

### Respiratory viruses and associated clinical features

The clinical diagnosis in order of frequency were rhinopharyngitis in 67%, pharyngitis in 18%, influenza-like illness, pharyngotonsillitis, and laringotracheitis in 3%, followed by bronchiolitis and rhinitis in 2%, rhinosinusitis in 0.6%, and pertussis-like syndrome in 0.2% (Table 3). All children were managed in an ambulatory fashion, and none required hospitalization. The most frequent signs and symptoms were cough in 85%, rhinorrea in 81%, nasal congestion in 70%, nasal discharge in 68%, fever in 68% and odynophagia in 61%. Other less frequent signs and symptoms were headache in 36%, myalgia in 25%, nausea in 21%, vomiting in 22%, dysphonia in 17%, respiratory distress in 13%, diarrhea in 11%, dyspnea in 11% and conjunctivitis in 8% of the children (Table 3).

**Table 3:** Clinical characteristics of children with respiratory tract infections in the community setting in the state of Veracruz

Clinical diagnosis	Córdoba	Tierra Blanca	Veracruz	Poza Rica	Minatitlán	Total
	n = 59	n = 70	n = 65	n = 210	n = 112	n = 516
Pertussis-like syndrome, n (%)	0	0	0	0	1 (1)	1 (0.2)
Rhinosinuisitis, n (%)	0	2 (3)	0	1 (0.5)	0	3 (0.6)
Bronchiolitis, n (%)	5 (9)	0	0	6 (3)	0	11 (2)
Rhinitis, n (%)	0	0	11 (17)	0	0	11 (2)
Laryngotracheitis, n (%)	6 (10)	0	4 (6)	3 (1.5)	1 (1)	14 (3)
Pharyngotonsillitis, n (%)	0	0	10 (15)	0	5 (4)	15 (3)
Inlfuenza-like illness, n (%)	0	17 (24)	0	0	0	17 (3.3)
Pharyngitis, n (%)	3 (5)	6 (9)	11 (17)	75 (36)	0	95 (18)
Rhinofaringitis, n (%)	45 (76)	45 (64)	29 (45)	125 (60)	105 (94)	349 (68)

Clinical characteristics	Córdoba	Tierra Blanca	Veracruz	Poza Rica	Minatitlán	Total
	n = 60	n = 77	n = 66	n = 210	n = 112	n = 525
Conjunctivitis, n (%)	2 (3)	4 (5)	16 (24)	16 (8)	5 (4)	43 (8)
Diarrhea, n (%)	9 (15)	11 (14)	2 (3)	28 (13)	7 (6)	57 (11)
Dyspnoea, n (%)	5 (8)	30 (39)	6 (9)	10 (5)	8 (7)	59 (11)
Respiratory distress, n (%)	6 (10)	30 (39)	0	16 (8)	18 (16)	70 (13)
Dysphonia, n (%)	9 (15)	14 (18)	15 (23)	29 (14)	20 (18)	87 (17)
Nausea, n (%)	12 (20)	13 (17)	0	76 (36)	10 (9)	111 (21)
Vomiting, n (%)	17 (28)	14 (18)	4 (6)	60 (28)	22 (20)	117 (22)
Myalgia, n (%)	4 (6)	7 (9)	4 (6)	103 (49)	13 (12)	131 (25)
Headache, n (%)	3 (5)	10 (13)	3 (4)	155 (74)	16 (14)	187 (36)
Odynophagia, n (%)	18 (30)	23 (30)	30 (45)	191 (91)	57 (51)	319 (61)
Nasal discharge, n (%)	46 (77)	54 (70)	41 (62)	109 (52)	105 (94)	355 (68)
Fever, n (%)	30 (50)	44 (57)	19 (29)	206 (98)	57 (51)	356 (68)
Nasal congestion, n (%)	44 (73)	68 (88)	48 (73)	132 (63)	77 (69)	369 (70)
Rhinorrea, n (%)	57 (95)	64 (83)	58 (88)	140 (66)	108 (96)	427 (81)
Cough, n (%)	54 (90)	63 (81)	53 (80)	184 (87)	95 (85)	449 (85)

We found differences in the pathogens detected among single viral infections according to the clinical diagnosis. In the children with rhinopharyngitis the most frequent pathogen was RSV A and B in 20%, followed by RV in 13.5%, FluA in 5.4%, AdV in 3.7%, and HMPV in 3.4%. In contrast, in children with pharyngitis the most frequent pathogens were RV in 10.5%, EV in 8.4%, FluA in 7.4%, HuCoV 229E/NL63 in 6.3%, and HMPV and PIV-3 in 4.2%. In children with rhinitis the pathogens detected were RSV-A in 27.3%, RV in 9.1%. The pathogens that affected the larynx, trachea and bronchiols were RSV, RV, and FluA (Table 4).

**Table 4:** Respiratory pathogens detected according to the clinical diagnosis

	Rhino pharyngitis	Pharyngitis	Rhinitis	Pharyngo tonsillitis	ILI	Rhinosinusitis	Laryngo tracheitis	Bronchiolitis	Pertussis-like syndrome
	n = 349	n = 95	n = 11	n = 15	n = 17	n = 3	n = 14	n = 11	n = 1
Adenovirus, n (%)	13 (3.7)^a^	2 (2.1)	ND	ND	1 (5.9)	1 (33.3)	ND	ND	ND
Bocavirus, n (%)	5 (1.4)	ND	ND	ND	ND	ND	ND	1 (9.1)	ND
HuCoV, 229E/NL63, n (%)	7 (2)	6 (6.3)	ND	ND	1 (5.9)	ND	ND	ND	ND
HuCoV OC43, n (%)	8 (2.3)	ND	ND	1 (6.7)	ND	ND	ND	ND	ND
Enterovirus, n (%)	9 (2.6)	8 (8.4)	ND	ND	ND	ND	ND	ND	ND
Influenza A, n (%)	19 (5.4)	7 (7.4)	ND	4 (26.7)	2 (11.8)	ND	2 (14.3)	ND	ND
Influenza B, n (%)	1 (0.3)	2 (2.1)	ND	2 (13.3)	1 (5.9)	ND	ND	ND	ND
Metapneumovirus, n (%)	12 (3.4)	4 (4.2)	1 (9.1)	ND	ND	ND	ND	1 (9.1)	ND
Parainfluenza 1, n (%)	6 (1.7)	2 (2.1)	ND	ND	ND	ND	ND	ND	ND
Parainfluenza 2, n (%)	2 (0.6)	1 (1.1)	ND	1 (6.7)	ND	ND	ND	ND	ND
Parainfluenza 3, n (%)	4 (1.1)	4 (4.2)	ND	ND	ND	ND	ND	1 (9.1)	ND
Parainfluenza 4, n (%)	4 (1.1)	1 (1.1)	ND	ND	ND	ND	ND	ND	ND
Rhinovirus, n (%)	47 (13.5)	10 (10.5)	1 (9.1)	1 (6.7)	ND	ND	2 (14.3)	2 (18.2)	ND
RSV A, n (%)	66 (18.9)	3 (3.2)	3 (27.3)	ND	4 (23.5)	ND	3 (21.4)	2 (18.2)	ND
RSV B, n (%)	4 (1.1)	1 (1.1)	ND	ND	ND	ND	ND	ND	ND
Negative, n (%)	94 (26.9)	34 (35.8)	4 (36.4)	4 (26.7)	6 (35.3)	1 (33.3)	3 (21.4)	2 (18.2)	1 (100)
Viral coinfection, n (%)	48 (13.7)	10 (10.5)	2 (18.2)	2 (13.3)	2 (11.8)	1 (33.3)	4 (28.6)	2 (18.2)	ND

*ILI* influenza-like illness, *ND* not detected.

^a^The numbers and percentage values refer only to viruses found in simple infections. Those found in coinfections are not considered in this table.

## Discussion

In this work viral pathogens were detected in a large proportion of children with respiratory disease (71.6%) as compared to studies previously carried out in Mexico. In a study conducted by the Epidemiologic Reference Institute (InDRE) of Mexico, 11% of the samples collected from different regions of the country between 1995 and 2000 were positive for viruses [7]. In hospitalized children, a respiratory virus was detected in 47% of patients with lower respiratory tract infections in a large referral Hospital in San Luis Potosi [8], and in 14% in the Hospital Infantil de México Federico Gómez [9]. In other studies carried out in the community setting, up to 65% of viral detection has been described [10–12], and the detection rate in secondary care hospitals has been reported to be between 39 and 60% [8, 11, 13].

RSV and RV were the most frequent pathogens detected in this work. Other studies carried out in Mexico have also reported RSV as the most frequent pathogen varying from 24 to 65% in hospitalized children with lower respiratory tract infections [8, 14] to 38% in a community setting [11], although in these studies RV was not looked for. In contrast, a recent report from Switzerland using molecular tests, described picornavirus as the most prevalent virus in upper respiratory tract infections in children (48%), followed by AdV (16%) and RSV (14%), although the population included in this study was different from those carried out in Mexico since it was performed in children hospitalized in a tertiary care center [15]. In another study carried out in Mexico in children younger than 18 years of age with influenza-like illness, influenza was the most frequent virus found (16.7%), followed by RSV (13.2%) and RV (11.2%) [16].

In this work HMPV was not detected as frequently as in Yucatán, in a study carried out in children with influenza-like illness, where 20% of the samples were positive for this virus [17]. Also, 10.5% of children hospitalized in San Luis Potosí, which had been negative for other respiratory viruses, were reported to be positive for HMPV [12]. In our study HMPV was found in 5.3% of the tested samples.

Some differences in the occurrence of viruses among municipalities were observed, with the highest viral diversity found in Poza Rica and Minatitlán, where all 15 searched viruses circulated; the frequency of the different viruses was more evenly distributed in Poza Rica. These two cities, located in the Northern and Southern part of the State of Veracruz, respectively, are centers of oil extraction, and they probably have a higher traffic of people than the other municipalities, what could explain the more ample variety of viruses found.

Multiple viral infections were detected in 13.9% of the samples. Detection of several viruses in a single sample is frequently reported in respiratory infections in which a PCR-based diagnostic method is used, however, the clinical relevance of detection of more than one virus is uncertain [18–21]. In this study, however, a tendency of correlation between coinfections and the severity of disease was observed. In children with laryngotracheitis and bronchiolitis, viral coinfection was detected in 28 and 18% respectively, compared to 13.8 and 10.5% of rhinopharyngitis and pharyngitis, respectively. This suggests that a viral coinfection could predispose to a more severe disease, although the low number of children in this study with more severe disease limits this conclusion. In addition, whether the detection of virus in nasal swabs reflects the actual pathogen of the clinical disease or reflects only nasopharyngeal carriage or an asymptomatic infection is not known, particularly when highly sensitive molecular methods are employed [22].

The seasonal predominance for RSV-A in autumn and winter, influenza in winter and spring, and HMPV from February to May observed in this study is consistent with previous reports, while RV, EV, AdV, and PIV were detected wit similar frequency along the study.

One limitation of this study is the great variability of the sample size in each city, since in Orizaba 60 patients were recruited, while in Poza Rica 208 were studied. For this reason, this study should be considered as descriptive, in which the statistics shown analyze the differences in the proportions within each city, while comparisons between cities were not done.

Many studies have described the frequency of viruses in hospitalized patients with severe respiratory illness, but studies describing the prevalence of different viruses in the community setting are limited, particularly in developing countries, where most of the deaths from serious respiratory diseases occur. This is the first report to evaluate a large panel of viruses in clinical samples from children with upper respiratory tract infections in the community setting in Mexico, and it provides recent information on respiratory viral pathogens not detected previously, and on the role each of them has as compared to the burden of other viruses.

## Conclusions

The availability of new molecular diagnostic methods with an increased sensitivity provides a more accurate depiction of the epidemiology of known pathogens and emerging respiratory viruses. However, although testing for common respiratory pathogens in children with acute respiratory tract infections may lead to a better understanding of the role of different viruses as etiologic agents, and eventually to improvement of individual patient management, additional prospective studies are required to study the need of routinely using such tests in general pediatric practices in resource-limited countries.

## Methods

### Study design

A prospective clinical, epidemiological, observational, cross-sectional study was conducted in Córdoba, Tierra Blanca, Veracruz, Poza Rica and Minatitlán, capital cities of five different municipalities of the state of Veracruz, Mexico. 525 Children with clinical signs and symptoms of respiratory infections attending the private practice with pediatricians of the Pediatric College of Veracruz were included. Nasal swabs were obtained from these children, samples were placed in REMEL M4-RT viral transport media, frozen at −20°C, and transported to the Instituto de Biotecnología, UNAM (IBt/UNAM), in Cuernavaca, Morelos, and stored a −70°C until analyzed. Clinical signs and symptoms, diagnosis, and risk factors were recorded for each patient in a format specially designed for the study. This study (project 186) was approved by the institutional review board of IBt/UNAM. Written informed consent was obtained from each parent or guardian prior to enrollment. The authors declare no conflict of interest.

### Detection of respiratory viruses

Nucleic acids were extracted from 200 µL of each sample using the PureLink^®^ Viral RNA/DNA Mini Kit following the manufacturer’s instructions. Extracted nucleic acids were eluted in 33 µL of RNase-free ddH2O and stored at −70°C until used. The Seeplex^®^ RV15 ACE Detection kit (Seegene Inc., Seoul, South Korea) was used to simultaneously detect 15 respiratory viruses: parainfluenza virus 1 (PIV-1), PIV-2, PIV-3, PIV-4, adenovirus A/B/C/D/E (AdV), human coronavirus 229E/NL63 (HuCoV), HuCoV-OC43, rinovirus A/B/C (RV), influenza A (FluA), influenza B (FluB), respiratory syncyctial virus A (RSV-A), RSV-B, human bocavirus 1/2/3/4 (HuBoV), human metapneumovirus (HMPV), and enterovirus (EV). Sample nucleic acids were tested following the protocol supplied by the manufacturer. PCR products were visualized by electrophoresis on a 2% agarose gels, staining with ethidium bromide.

### Statistical analysis

Univariate analysis was used to determine frequencies and proportions. For bivariate analysis Kruskal–Wallis test was used to contrast qualitative variables and ANOVA for quantitative variables. Multivariate analysis was performed to detect correlation among clinical features, clinical diagnosis, risk factors, municipalities, and viral or bacterial detection. A p < 0.05 was considered statistically significant.

### Consent

Written informed consent was obtained from each parent or guardian prior to enrollment in the study.
